# Creating, generating and comparing random network models with NetworkRandomizer

**DOI:** 10.12688/f1000research.9203.3

**Published:** 2017-11-10

**Authors:** Gabriele Tosadori, Ivan Bestvina, Fausto Spoto, Carlo Laudanna, Giovanni Scardoni

**Affiliations:** 1 Department of Computer Science, University of Verona, Verona, 37134, Italy; 2 Center for BioMedical Computing, University of Verona, Verona, 37134, Italy; 3 Faculty of Electrical Engineering and Computing, University of Zagreb, Zagreb, 10000, Croatia; 4 Department of Medicine, University of Verona, Verona, 37134, Italy

**Keywords:** random networks, cytoscape, network randomisation, network analysis, network generation

## Abstract

Biological networks are becoming a fundamental tool for the investigation of high-throughput data in several fields of biology and biotechnology. With the increasing amount of information, network-based models are gaining more and more interest and new techniques are required in order to mine the information and to validate the results. To fill the validation gap we present an app, for the Cytoscape platform, which aims at creating randomised networks and randomising existing, real networks. Since there is a lack of tools that allow performing such operations, our app aims at enabling researchers to exploit different, well known random network models that could be used as a benchmark for validating real, biological datasets. We also propose a novel methodology for creating random weighted networks, i.e. the multiplication algorithm, starting from real, quantitative data. Finally, the app provides a statistical tool that compares real versus randomly computed attributes, in order to validate the numerical findings. In summary, our app aims at creating a standardised methodology for the validation of the results in the context of the Cytoscape platform.

## Introduction

In network analysis many tools have been developed to address different problems related to the extraction of useful information from systems modelled as graphs ^1^. Cytoscape ^2^ is a very well known platform, supporting hundreds of apps, that simplifies the information mining in complex networks, with a specific focus on biological applications. By using Cytoscape it is possible to perform topological analysis, cluster and motif retrieval, biological enrichment, draw nice graphs, search for ontologies, etc. As the number of apps increases, the possibilities of performing more and more complex analysis grows together with the amount of information that could be used and retrieved. The problem is that this analysis remains, in general, preliminary to further experimental validations and, in this sense, a sort of benchmark for an *in-silico* validation is required ^3^. A possible solution to this issue may come from the biological background of the process, from the literature or from experimental data ^4^. But this is not always possible since the network may represent some complex processes whose biology is yet not well understood, or that take advantage of some novel insights that require a different validation. In these cases, when biological evidences are missing or incomplete, an interesting approach allows comparing the real experiments with a set of randomly generated experiments to verify the robustness of the real data ^5^. In network analysis this is achieved by comparing the networks under investigation, with some randomly generated networks.

It is important to note that some apps that allow performing network comparison exist. For instance DyNet ^6^, GASOLINE ^7^, NetMatch* ^8^, and RandomNetworks ^9^. RandomNetworks has a lot of similarities with our app but is no longer maintained and it works only with Cytoscape 2.x which makes it useless with the newly developed Cytoscape platform. This was one of the main reasons that inspired NetworkRandomizer. The other apps we cited allow comparing network but none of them allows generating random networks.

In this sense, our app was created to address the problem of creating a validation layer that allows simulating random experiments. By using randomly created networks, it becomes possible to compare, by means of specific statistical tests, the numerical results that come from a common network analysis. To do so, NetworkRandomizer allows randomizing existing networks by using a simple shuffling algorithm and a degree preserving algorithm. Moreover it allows creating Erdős–Rényi, Barabási–Albert, Watts-Strogatz, Lattice, and Community Affiliation models. We also implemented the Multiplication model which is designed to generate weighted networks where nodes are multiplied, i.e. represented in different copies which have the same topological characteristics, to fit quantitative data. Finally a statistical module, based on the two-sample Kolmogorov-Smirnov test, compares networks in order to evaluate if their numerical attributes come from the same distribution or if they should be considered different. The original idea behind the app was to compare topological indexes, the so called centralities, computed by using CentiScaPe ^10^ or the Cytoscape’s built-in Network Analyzer. However, our app supports the comparison of all kinds of numerical attributes in order to be useful to all users. It is important to note that comparing random data with real data is not always a trivial operation. Generating random networks does not necessarily mean that we are able to obtain actual random networks. Indeed, some basic information, like for instance the number of nodes or edges, will always make the randomisation non random at some levels. Also we do not know which way is better in creating random networks in order to compare them with real data. Finally, it is important to consider that, when creating an *in-silico* dataset for validating real data, the bigger the random dataset, i.e. the more the networks, the finer the comparison will be. Eventually, the results will be more reliable.

To make an example that clarifies the utility of our app, lets start with a network that models a specific pathology, i.e. the protein-protein interactions that are known to be involved in the development of lung cancer. Lets suppose that, by performing a network analysis, some interesting properties arise like, for instance, a very peculiar centrality index, e.g. Eccentricity. It could be interesting to build a set of randomised networks, that comprise the same number of nodes and edges as the original, biological network, in order to evaluate if the Eccentricity values are found in randomised networks or if it is a very specific, hence important, property or our network. By using our app, performing such comparison becomes very easy and accessible through Cytoscape.

## Methods

### Implementation

NetworkRandomizer follows a modular structure, making it easy to add additional random network models in future releases. Each model is initialised with some user’s specified parameters and once the generation is done the app deploys the network into the Network Control Panel, making it instantly available to the user for future use. Since the user may want to generate a number of random networks, the network views are not created by the app, in order to avoid pop-ups of networks in the Cytoscape window during the algorithm’s computation.

The app is divided in several classes which allow a very flexible and modular structure. The CyActivator class runs the application and communicates with Cytoscape. The RandomizerCore class is used as a model of the current Cytoscape state (network handling etc.). The MenuAction class allows app initiation. The OptionsMenu class refers to the main GUI which is used to interact with the app. The ThreadEngine allows creating and handling threads and multithread tasks. Finally the AbstractModel is an abstract class which defines the basic random model and offers several methods that could be useful when defining a new model. The other classes in the app refer to the models we implemented. In order to add a new model, a new class should be instantiated by following the AbstractModel implementation. Then it is necessary to modify the GUI in order to let the new model become part of the app.

The statistical module that we implemented is a two-sample Kolmogorov–Smirnov test ^11^. It takes each pair of real and random network, and for each of the attributes used, it calculates the difference between their distribution. The K *−*S definition of the distribution difference is the maximum gap between the cumulative probability functions of those probabilities. Although it relies on the cumulative probability functions, they never need to be explicitly calculated. In fact, the algorithm only needs to sort the two lists of attribute’s values and then run through them in parallel, summing the normalised (divided by the sum) values along the way and comparing the two sums, saving only the largest difference.

### Operation

**System requirements.** To install and run the NetworkRandomizer app, the Cytoscape software must first be installed. Once this is done, no additional system requirements exist since the app does not use any external library nor does it significantly increase the memory consumption. An updated Java version is suggested, since the last Cytoscape version works only with Java 8.

It is important to note that, when using the Multiplication model, the number of nodes that are generated could result in a huge network. This is due to the model itself which, starting from an user defined attribute, creates an array of random weights which will result in a number of nodes. It works by creating a range between the minimum and the maximum values found in the file, hence in the worst case the algorithm will create a network with *#max* copies of nodes for each node. In numbers this means that if the attribute file varies in a range [0−100] and the real network is made of 10 nodes, then the algorithm in the worst case may generate a network with 100 ∗ 10 nodes. Memory usage problems may also arise within the other models, depending on the number of nodes the user selects for creating a random network.

**Workflow.** The usual NetworkRandomizer workflow consists of a few distinct steps. First of all, the user needs to load one or more real networks. Depending on whether one or multiple networks are loaded, the final output will be different. It consists of a file which shows the results of the statistical analysis. If there is only one real network, the output file will present more details about it. If there are multiple real networks, then the output file will be a summary of all the statistical tests that were carried out.

After loading the networks, the randomisation step creates random networks. The random networks may be created either by randomizing real networks, using the edge shuffle algorithm (degree preserving or completely random) ( Figure 2), or by generating new random networks by using one of available models ( Figure 3 and Figure 4).

After real networks are loaded and random networks generated, it is possible to compare their shared attributes. Since the app was initially designed to be used in conjunction with centrality parameters, we recommend using the CentiScaPe app which is completely compatible with our NetworkRandomizer. It is important to note that the app allows comparing the attributes with the same name in all the networks that are selected within the statistical module. Basically, once the networks are selected, only the shared attributes across all networks would appear as possible attribute choices. If there are no attributes that are shared, the user should check the names in the Node Table panel of Cytoscape.

**Figure 1.**
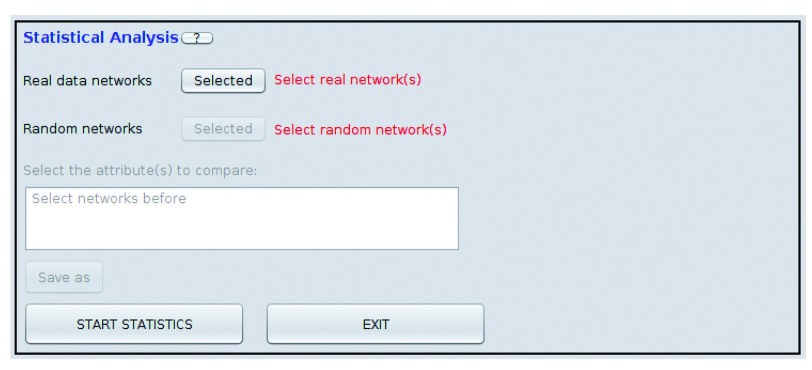
Statistical module: after data are generated, the statistical module allows comparing all the networks attributes in order to find important patterns. The attribute to be compared must have the same name in all the networks that are selected (it is also case-sensitive).

Finally, once all the data is available, the statistical test ( Figure 1) compares the selected attributes, pinpointing their differences and similarities and giving a textual file which summarises the results. Each part of the app has a question mark button which provides help to the users, making the NetworkRandomizer easier to use, as well as appropriate for educational purposes.

**Figure 2.**
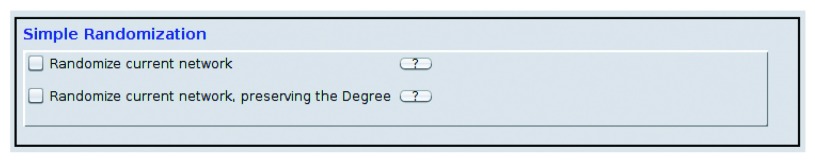
Randomisation interface: the users can choose between a simple edge shuffle, or the degree preserving version. Both of them are intended for randomizing an already existing networks.

**Figure 3.**
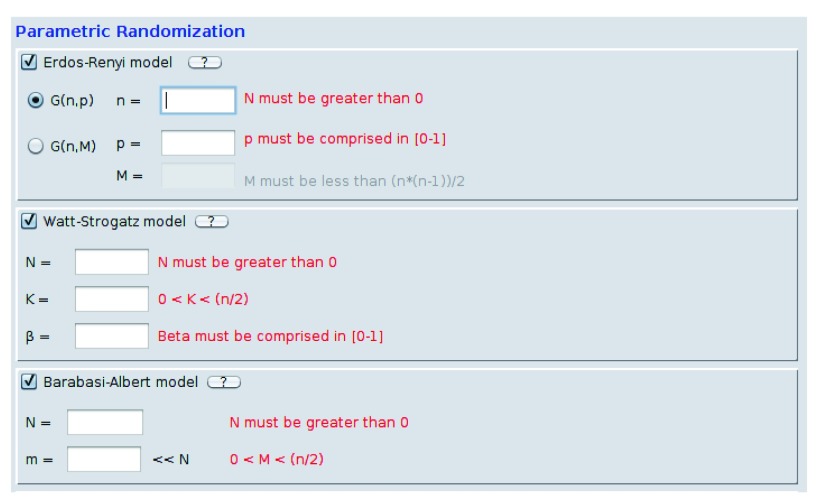
Random network models: Erdős–Rényi, Watts–Strogatz, Barabási–Albert. They require some parameters which are inserted by the user. Some labels, in red, help the user in order to correctly fill the fields.

**Figure 4.**
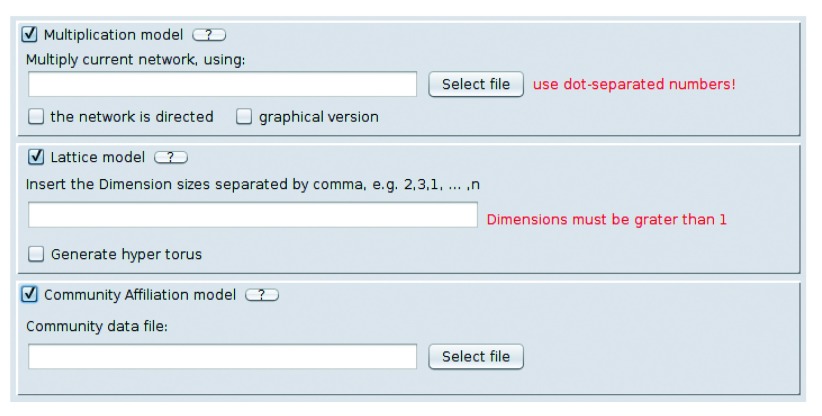
Multiplication model network generator, lattice generator and Community Affiliation Graph model. The Multiplication and the Community Affiliation requires a file as input, in order to generate random networks.

## Use cases

**Data preparation.** In order to use the app we present a typical use case to guide the users through the network analysis process, using the NetworkRandomizer.

The first step when analyzing any network with Cytoscape apps is to import the network into the Cytoscape software. After that, as said, some information is required, and even though we focused on topological centralities, it is possible to use any kind of numerical information. There are multiple ways to fill in this information. It is possible to import a *.csv* file, to create a new attribute or to use a built-in network analyzing function.

A more advanced approach is to use a popular centrality measures app, i.e. CentiScaPe, which can be downloaded from the Cytoscape Apps store. It provides the users (and consequentially the NetworkRandomizer itself) with more information about the networks, and, finally, results in a much wider perspective on differences between them. It is important to note that by using these apps one obtains standard names for the attributes. Caution should be taken when using user defined attributes, that the same attribute names are used for the real and for the random networks otherwise no attribute will appear while performing the statistical analysis.

**Generating random networks.** There are two main methodologies for generating random networks. By using the random network models or by randomizing current, real networks. For obtaining better results, random networks should be made as similar as possible to the real ones, as this will allow detecting the most important differences between those two groups.

To randomise an existing network, one needs to be chosen first. This is done by simply creating a View: right click on the network from the Network Control Panel, and choose Create View. Once the network is selected, there are two randomizing methods that can be used: simple edge shuffle, and degree preserving edge shuffle. Users can choose one or both models by checking the boxes ( Figure 2). Once the module is selected, the Start button runs the randomisation.

Apart from the randomisation option, the main part of the Randomizer consists of multiple random network models. Currently implemented models are: Erdős–Rényi ^12^, Watts–Strogatz ^13^, Barabási–Albert ^14^, lattices and Community Affiliation Graph ^15^. There is also a new model which we called the Multiplication model.

Erdős–Rényi model ( Figure 3) generates fully random networks by either uniformly choosing *M* pairs of nodes to connect, or by connecting each pair with probability *p*. Watts–Strogatz model ( Figure 3) generates networks which show the so called small-world phenomenon. Here, although the network is not dense, the average shortest path is still significantly low. Networks generated by using the Barabási–Albert model ( Figure 3) are scale-free networks, meaning that their degree distribution follows a power law distribution. The model uses the preferential attachment growth, hence each new node is connected to *m* other nodes, choosing them with a probability which is proportional to their degree. Once a model is selected the users are informed about the parameter constraints by a red label ( Figure 3). The labels update their values, in order to be more helpful, once they are filled in correctly and after pressing the Enter button.

**Figure 5.**
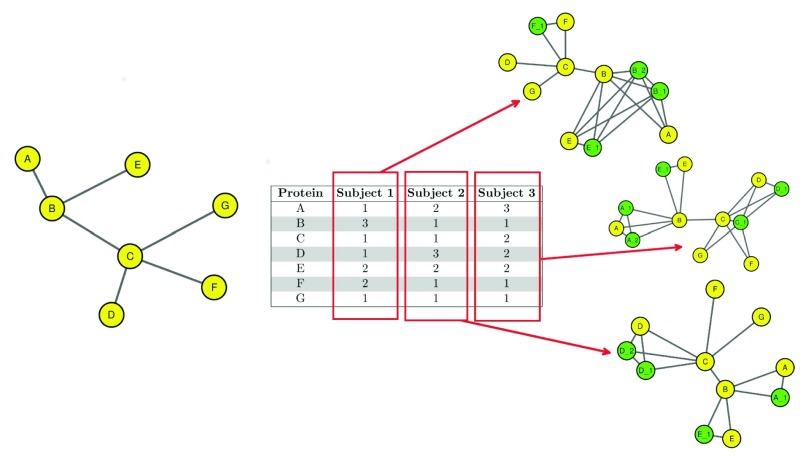
The algorithm for the multiplication of the nodes, starting from the network on the left, creates a random array for each new random network that will be generated. By using the values in the random array a number of nodes, in green, are added to the original network, represented by the yellow nodes and some new networks are created.

Multiplication model ( Figure 4) generates randomly weighted network ( Figure 5). The algorithm generates a random array which defines a weight for each node belonging to an existing, user-defined network, starting from an attribute file which contains quantitative information about the nodes. The file may contain two or more numerical values defining the quantitative values represented by a possible experimental setup. The algorithm creates a range extracting from this file the minimum and the maximum values and then generates a number of random weights in the range that are assigned to the nodes. Finally the algorithm creates a new network which contains the same number of copies, but with a new attribute that defines the weight that are assigned to each node. This random weighted network represents a possible experimental setup which derives from the attribute file. In this sense it is possible to generate a number of randomly weighted networks, in order to simulate a set of experiments. The new nodes, that are added as copies of existing nodes, have the same neighbours of the original node and share an edge with the original node and each of its copies. The multiplication model gives two different outputs. The default output is the network that is being multiplied with a new attribute that defines the new, randomly assigned weights. The second, user selected, output is a graphical version of the network showing the actual number of nodes, depending on their weights. It is important to note that the file that is passed as input should contain numerical values, separated by a dot in case of floating values. Also it is important to note that, by passing a file with only zero or one the returned network will not be multiplied since the algorithm generates a range that is in between zero and one. To properly multiply a network the input file should contain values higher than one.

The *graphical version* option in the multiplied model tab ( Figure 4) allows, if selected, to obtain a network which actually shows the new copies of each multiplied node. This network is not intended for computing centralities but for graphical purposes only. If the option is selected another network that does not shows the copy of the nodes is generated.

Lattice model ( Figure 4) generates regular, multidimensional lattices. Multidimensional lattices are grids of nodes, each of which is connected only to its first neighbours. For example, the one-dimensional lattice is a path graph and is obtained by inserting *n* = 1 value (for example "5" for a chain of length 5), while the two-dimensional lattice is a square grid, obtained by inserting *n* = 2 values (for example "3,4" for a 3 by 4 grid). Additionally, users have the option of generating torus-shaped lattices, where there are no endnodes. For example, the one-dimensional torus lattice is a cycle graph, two dimensional torus lattice is a three dimensional torus, etc.

Finally, the Community Affiliation model ( Figure 4) generates random networks from the community information: given a list of communities, their members, and the probability of having an edge between two members of each community, it randomly generates realistic social networks.

Some models require special input files which define their behaviour. The two models that currently require an input file are *Multiplication* and *Community Affiliation*. The Multiplication file must contain numerical values, one for each row. It is expected, but not mandatory, that the number of rows is equal to the number of nodes and the Randomizer will pop up a dialog which asks the user if the number of values found in the file is correct. The Community Affiliation model requires a file which contains a set of rows where each line represents a community. The rows starts with a p-value - the probability of an edge between two members of the community, and is followed by the names of the nodes inside that community, separated by spaces.

**Figure 6.**
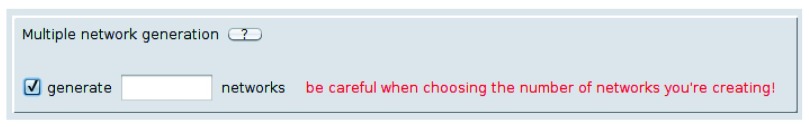
This form allows the user in order to choose how many networks will be generated, once the Randomizer runs, for each selected model.

One last point concerns the fact that, once the model is selected in the app panel, every time the button Start Randomisation is pressed, each selected model is created in a number of copies chosen by the user ( Figure 6. If the number of networks is not specified, by using the form, the algorithm will generate one network per model. Otherwise the selected number of networks will be generated, for each selected model. In other words, if the user select the two randomisation algorithms, i.e. *Degree preserving* and *Randomize Current Network* and then runs the app, as a result the app will return two randomised network. This means that, after a computation, we suggest removing the check mark from all the models in order to avoid creating a number of unrequired new networks, every time NetworkRandomizer runs.

**Comparing networks.** Once random networks are generated, before comparing their attributes to the real ones, at least one numerical attribute should be present in all the networks that will be compared. To run the statistical comparison module, it is necessary to specify which networks it will use. To do this, users need to select the real networks in the Networks Control Panel and press Selected, and then do the same for the random ones. After selecting the networks, a list of all the node attributes shared within all the selected networks is provided. Users can now select the node attributes they would like to compare. If there is not a shared attribute then a dialog will appear telling the user to check the attributes again. Selection is done using the left-click and additional keys, *Ctrl* for one-by-one multiple selection, *Shift* for range selection, and *Ctrl+A* to select all. Once the attributes are selected, output file name and directory need to be specified, and the comparison can be executed by clicking the Start Statistics button.

**Interpreting the results.** Every output file consists of multiple comma-separated-values fields, each one beginning with an explanation marked by the **>** symbol. First few fields define the names of the networks used. The other fields differ, depending on whether only one, or multiple real networks are used.

If there is only one real network, output is either fragmented into centrality measures used, or into random networks to which the real one is compared. The first field indicates how different is each random network from the real one by using the average difference across all centralities. The second field represents the difference between the real network and its most similar random network, according to each centrality measure individually. The last field provides more in-depth information, specifying the difference between the real network and each random one, for each centrality measure.

Showing all of the generated data to the user, in the case of multiple real networks, would result in a very unreadable output. To avoid this, only the most interesting points are chosen: either the pairs of real and random networks, or the pairs of real networks and centrality measures which show the least statistical differences. This way, users can check their networks for important non-random processes. The first field specifies the average difference across all centralities between real networks and their most similar random network. The second field shows the most similar random network with respect to the real one, for each real network and each centrality, and specifies their difference. The last field is the real-random distance matrix, with distances defined as average difference across all centralities. A value of 0 indicates that the distributions are completely the same, up to a normalisation factor. Normalisation factor, in this sense, is the number of elements in the series. So, for example, the series [1, 2, 3] and [1, 1, 2, 2, 3, 3] would be completely the same. A value which is close to 1 indicates the existence of an important difference between the distributions. This result is rarely achieved in real datasets, and it happens when the elements of one series are all greater than the elements of the other.

## Summary

To summarise, our app allows generating and creating random networks and is useful whenever a validation benchmark is required. Starting from real networks it is possible to compare their attributes with randomly generated attributes obtained by analysing random networks. The main issue concerns the fact that there is not a specific model that should be suggested in a specific setup or with some specific kinds of data. It is up to the user to select which model best describes the network and its mathematical characteristics.

Concluding, our app is designed to be as general as possible having a very wide range of applications and to be completely user-friendly, giving the possibility to perform a simple, but meaningful, statistical analysis and a readable output.

## Software availability

The NetworkRandomizer can be downloaded at: http://apps.cytoscape.org/apps/networkrandomizer

Source code is available at: https://github.com/gabrielet/Network-Randomizer

Archived source code as at time of publication: http://doi.org/10.5281/zenodo.159271 ^16^

Software license: Apache License, Version 2.0

## References

[1] Saito R Smoot ME Ono K: A travel guide to Cytoscape plugins. *Nat Methods.* 2012;9(11):1069–1076. 10.1038/nmeth.2212 23132118 PMC3649846

[2] Cline MS Smoot M Cerami E: Integration of biological networks and gene expression data using Cytoscape. *Nat Protoc.* 2007;2(10):2366–2382. 10.1038/nprot.2007.324 17947979 PMC3685583

[3] Sah P Singh LO Clauset A: Exploring community structure in biological networks with random graphs. *BMC Bioinformatics.* 2014;15(1):220. 10.1186/1471-2105-15-220 24965130 PMC4094994

[4] Haibe-Kains B Emmert-Streib F: Quantitative assessment and validation of network inference methods in bioinformatics. Frontiers Media SA, *Front Genet.* 2014;5:221. 10.3389/fgene.2014.00221 25076966 PMC4099936

[5] Newman ME Watts DJ Strogatz SH: Random graph models of social networks. *Proc Natl Acad Sci U S A.* 2002;99(suppl 1):2566–2572. 10.1073/pnas.012582999 11875211 PMC128577

[6] Goenawan IH Bryan K Lynn DJ: DyNet: visualization and analysis of dynamic molecular interaction networks. *Bioinformatics.* 2016;32(17):2713–5. 10.1093/bioinformatics/btw187 27153624 PMC5013899

[7] Micale G Pulvirenti A Giugno R: GASOLINE: a Greedy And Stochastic algorithm for optimal Local multiple alignment of Interaction NEtworks. *PLoS One.* 2014;9(6):e98750. 10.1371/journal.pone.0098750 24911103 PMC4049608

[8] Rinnone F Micale G Bonnici V: NetMatchStar: an enhanced Cytoscape network querying app [version 2; referees: 2 approved]. *F1000Res.* 2015;4:479. 10.12688/f1000research.6656.2 26594341 PMC4642848

[9] Randomnetworks cytoscape app. [Online]. (Date last accessed 13-March-2017). Reference Source

[10] Scardoni G Petterlini M Laudanna C: Analyzing biological network parameters with CentiScaPe. *Bioinformatics.* 2009;25(21):2857–2859. 10.1093/bioinformatics/btp517 19729372 PMC2781755

[11] Wilcox RR: Some practical reasons for reconsidering the Kolmogorov-Smirnov test. *Br J Math Stat Psychol.* 1997;50(1):9–20. 10.1111/j.2044-8317.1997.tb01098.x

[12] Erdős P Rényi A: On Random Graphs. *Publ Math Debrecen.* 1959;6:290–297. Reference Source

[13] Watts DJ Strogatz SH: Collective dynamics of ‘small-world’ networks. *Nature.* 1998;393(6684):440–442. 10.1038/30918 9623998

[14] Barabasi AL Albert R: Emergence of scaling in random networks. *Science.* 1999;286(5439):509–512. 10.1126/science.286.5439.509 10521342

[15] Yang J Leskovec J: Community-affiliation graph model for overlapping network community detection.In *Data Mining (ICDM), 2012 IEEE 12th International Conference on*IEEE.2012;1170–1175. 10.1109/ICDM.2012.139

[16] gabrielet Bestvina I: F1000Research/Network-Randomizer: F1000Reserach/Network-Randomizer (Version v1.1). *Zenodo.* 2016, October 4. Data Source

