# Supplementary material for: Creating, generating and comparing random network models with NetworkRandomizer
Source: F1000Res. 2017 Nov 10;5:2524. Originally published 2016 Oct 17. [Version 3] doi: 10.12688/f1000research.9203.3 (PMC5686481; doi:10.12688/f1000research.9203.3)
Supplement: Peer review history

# Peer review: Referee response for version 3

**DOI:** 10.5256/f1000research.14219.r17035

**Published:** 2018-05-08

**Authors:** Ankush Sharma

**Affiliations:** 1 Department of Molecular Genetics, Erasmus Medical Centre, Rotterdam, Netherlands

Tosadori et al. have developed Cytoscape app that was much needed in the Cytoscape app store for comparing networks with Random networks with degree or other centrality statistics preservation. The updated version of research article deals with all the concern raised by the fellow reviewers.

 The manuscript is well written and work flow is easy to understand.

 Minor concerns:

1. I would suggest authors include a figure showing how centrality statistics are affected on randomizing networks with degree preservation (or any other centrality statistic preservation).
2. I would suggest authors use an appropriate word in place of "the so-called" as it is giving an impression that word "small world" and "centralities" are inappropriate.

I have read this submission. I believe that I have an appropriate level of expertise to confirm that it is of an acceptable scientific standard, however I have significant reservations, as outlined above.

# Peer review: Referee response for version 3

**DOI:** 10.5256/f1000research.14219.r27838

**Published:** 2017-11-10

**Authors:** Giovanni Micale

**Affiliations:** 1 Department of Math and Computer Science, University of Catania, Catania, Italy

Thanks for addressing all my last concerns about the paper and the software.

 I definitely approve this paper.

I have read this submission. I believe that I have an appropriate level of expertise to confirm that it is of an acceptable scientific standard.

# Peer review: Referee response for version 2

**DOI:** 10.5256/f1000research.12878.r24026

**Published:** 2017-07-04

**Authors:** Giovanni Micale

**Affiliations:** 1 Department of Math and Computer Science, University of Catania, Catania, Italy

Thanks for the efforts in revising the paper and the software according to my points.

 Almost all my points were addressed by you. In particular the Multiplication Model is now more clearly explained in the paper and also some obscure parts in the Cytoscape app were properly explained.

 I have still few concerns about the paper, that I report in the following:

1. The Google Doc link to the documentation of the app in the web page of NetworkRandomizer in the Cytoscape App Store seems to be broken. I had to download the manual from the gitHub page of the software. Please fix the link.
2. Concerning point 5 of my first review, you mentioned NetMatch*, GASOLINE (please change GASO-LINE to GASOLINE in the paper) and DynNet, but you forgot to insert references for these apps. Please add the references. For GASOLINE, please also cite the main paper ( http://journals.plos.org/plosone/article?id=10.1371/journal.pone.0098750), not just the cytoscape app paper.
3. Concerning point 11, I need to better explain my observation. Suppose that I complete a first statistical experiment with some selected input networks, some selected random networks and one or more selected attributes from the list. Now, suppose that I would like to make another experiment by changing only the attributes. In such a case I need to start again from scratch, by selecting input networks and random networks and finally the new attributes. So, every time I want to make a new statistical experiment I need to start from scratch. This can be tiring for the user. You should somehow fix it.
4. Anyway, I also observed that the red text near "Selected" buttons is always visible, even though I haven't still selected any network and even in the case I want to make a new experiment. I would suggest to show red texts only after the user has selected some networks, and clear them off whenever the user wants to make a new experiment. Moreover, I would also change the text of the buttons from "Selected" to "Add selected networks" to make the meaning of the button clearer.

As soon as these points will be addressed, I will definitely approve the paper.

I have read this submission. I believe that I have an appropriate level of expertise to confirm that it is of an acceptable scientific standard, however I have significant reservations, as outlined above.

# Peer review: Referee response for version 1

**DOI:** 10.5256/f1000research.9906.r17030

**Published:** 2016-11-01

**Authors:** Francesco Russo

**Affiliations:** 1 The Novo Nordisk Foundation Center for Protein Research, University of Copenhagen., Copenhagen, Denmark

The authors proposed a new Cytoscape App to generate random networks and compare them with real data. It consists of the most common approaches for network randomisation. Moreover, the authors proposed a novel method to generate random weighted networks.

 There are few implemented apps available in Cytoscape for this purpose, so this app will be very useful and simple to use in a well known framework.

 The manuscript is well written and clear. It could be nice and useful to have a figure representing the workflow of the proposed app, if it is compatible with the number of figures allowed for this journal.

I have read this submission. I believe that I have an appropriate level of expertise to confirm that it is of an acceptable scientific standard.

# Peer review: Referee response for version 1

**DOI:** 10.5256/f1000research.9906.r17046

**Published:** 2016-10-31

**Authors:** Giovanni Micale

**Affiliations:** 1 Department of Math and Computer Science, University of Catania, Catania, Italy

In the paper, Tosadori et al. describe a Cytoscape app for creating random networks according to different random models and comparing them with one or more input networks on the basis of different topological metrics.

 The main goal of the app is to help the user in the creation of a benchmark for validating some properties of a real network or a dataset of real networks.

 I found the app very interesting and I think that the paper is suitable for being indexed. However, there are several major issues, especially regarding the potential applications of the tool and the statistical part of the app, that need to be addressed before indexing.

1. The actual usefulness of the app is not fully explained. In the Abstract and in the Introduction the authors point out that the app should serve as a framework to validate some numerical results of one or more input networks, but it is not clear how the results of comparing values of topological attributes of real and random networks can be exploited by the user to draw conclusions. I would suggest to insert (for example in the Introduction) one or more examples of usage of this tool. For instance, an interesting application of the tool could be the following: generate different random networks (one or more for each random model) with a similar number of nodes and edges of the real network, then compare these networks with the real one according to some statistics (for example clustering coefficient) in order to find the random model that best fits the input network. This can tell a lot about the features of the network and how it has been generated.
2. The statistical module of the app allows to compare two distribution of values on some topological property of the nodes (for example centrality) by using the two-sample Kolmogorov-Smirnov test. The aim of this comparison should be carefully clarified. Is the aim to establish if topological properties are maintained with respect to the random model used? Does it make sense when such a value is obtained comparing only a pair of networks, the real and the random ones?
  Let’s consider a set of N networks generated using the preferential attachment and compare them to a real network according to the values of some metric M. The reviewer expects that will be sensible variations on the Kolmogorov-Smirnov distance. It would be more reasonable to give the user the possibility to compare expected values of some metric in ensembles of real and random networks. In some context, this kind of analysis can be preferable and would result more robust. 
3. Among the random models, the authors presented a new model called Multiplicative Model. It is not clear from the text how such a model works. There is a picture in the paper that shows three networks generated with three different weighted arrays, but it is not clear how these weighted arrays are generated. Which are the input parameters of the model? Please clarify how the weights are determined. It is not clear i) if the user has to provide a minimum value X and a maximum value Y and then weighted arrays are generated by randomly picking one value within interval [X,Y] for each node or ii) if just has to provide a text file with one weight for each node and generate a random network with exactly that sequence of weights. By using the app, it seems that a text file is required but it is not clear what should be its content. In general, authors should describe more clearly the model both in the paper and in the documentation of the app. 
4. It looks like the Multiplication model is not properly working: I tried to generate a random network using the Multiplication Model starting from an input network of N nodes. As a parameter for the model, I loaded an input file with N rows and one number for each row (in each row there is 0 or 1 as weight). A window appeared saying which was the maximum number of edges admitted for the network. However, at the end of the process, a random network with exactly the same number of nodes and edges (maybe a copy) of the input network was created without any error or warning message. I expected as output a network with a different number of nodes and edges. Please clarifiy this point. 
5. In the Abstract and in the Introduction authors claim that there are no tools for generating and randomizing networks. Actually, there are two Cytoscape apps that authors should cite in the paper: Randomnetworks plugin ( http://apps.cytoscape.org/apps/randomnetworks) and NetMatch* ( http://alpha.dmi.unict.it/netmatchstar/netmatchstar.html). Furthermore, since your app implements a comparison part between networks, I would also recommend to cite some other Cytoscape app to compare networks, such as GASOLINE ( http://apps.cytoscape.org/apps/gasoline) and DyNet ( http://apps.cytoscape.org/apps/dynet), remarking the different kind of approach to compare networks that you are following, which is the new research contribution. 
6. On page 6, third paragraph, when authors talk about the two shuffling methods (with or without preservation of degrees) they say that users can choose one or both models. Authors should describe which is the effect when both options are selected. 
7. What is the maximum number of nodes in a network generated with the multiplication model? In the example at page 3 for a network of 10 nodes and weights in [0,100] is 100*10+10, but in the figure of page 6 it seems to be just the number of nodes multiplied by the maximum value of the range (in the figure of page 6 it seems 7*3 and not 7*3+7). Please clarify this point. 
8. In the paper there is no indication of which sizes represent the Lattice Model (Figure 4). I finally deduced this information from the documentation. This should be reported with a sentence also in the main paper. 
9. There is a difference between the panel of Multiplication Model depicted in Figure 4 and the Multiplication Model panel present in the Cytoscape App. In the app I found an option called “Graphical version” which is not reported in the paper. Please clarify and make the app and the paper coherent on such a point. 
10. When the user generates random networks or run the statistical analysis there is no indication on the status of the computation. It would be recommended to notify the user with logs and progress bars. 
11. I tried to execute a pipeline of statistical experiments with different input networks and/or random networks. However, after the first experiment, it was impossible to me to change the set of selected input or random networks for a second experiment. I had to close and open again the app to make other tests. Please fix this bug. 
12. In the Statistical analysis panel there is a “Save as” button to select the path where to save output results. However, there is no text field next to the button that indicates the selected output folder. Please insert such a field.

I have read this submission. I believe that I have an appropriate level of expertise to confirm that it is of an acceptable scientific standard, however I have significant reservations, as outlined above.

# Response

**Published:** 2017-06-12

**Authors:** Gabriele Tosadori

**Affiliations:** Università degli Studi di Verona, Italy

Dear Giovanni,

Thank you for all the very useful comments. We amended the paper as you suggested and we hope it is now more clear and useful.

I would like to answer you with respect to the points 10, 11 and 12.

Point 10: we decided not to add the progress bar since the computation takes a very little time and we believe it is not a necessary feature. If you really think that it is needed, then we will add it for our newest release otherwise, we'll keep the app as it currently is

Point 11: we are not able to obtain the same bug you're mentioning. Please give us more feedback about this issue and we will see how to fix this.

Point 12: we decided not to add the path to the folder since it was creating a lot of issuse with the Mac version of the Java library. In order to avoid bugs for the Mac users we removed this feature.

If you have further questions and suggestions, we will be very happy to hear everything.

Thank you,

Gabriele Tosadori
